# Differentiating respiratory syncytial virus pneumonia from interstitial lung disease associated with systemic sclerosis: A case report

**DOI:** 10.1097/MD.0000000000045924

**Published:** 2025-11-14

**Authors:** Hiroki Wakabayashi, Kaichi Kaneko, Eisuke Tanaka, Ryogo Ohashi, Kensuke Namba, Misa Iwayanagi, Hiromasa Sakurai, Yusuke Irie, Daiki Sakai, Kenta Takashima, Yu Murakami, Ai Nishida, Kazutoshi Isobe, Yasuo Matsuzawa

**Affiliations:** a Department of Internal Medicine, Division of Respiratory Medicine, Toho University Sakura Medical Center, Sakura, Chiba, Japan; b Department of Internal Medicine, Division of Rheumatology, Toho University Sakura Medical Center, Sakura, Chiba, Japan.

**Keywords:** interstitial lung disease, pneumonia, respiratory syncytial virus, systemic sclerosis

## Abstract

**Rationale:**

Respiratory syncytial virus (RSV) is a common cause of lower respiratory tract infections in older adults. In systemic sclerosis, interstitial lung disease is a frequent, serious complication requiring immunosuppressive therapy. Differentiating RSV pneumonia from systemic sclerosis-associated interstitial lung disease (SSc-ILD) is essential for appropriate management and to avoid initiating immunosuppressive agents that may be harmful in cases of RSV pneumonia.

**Patient concerns:**

An 85-year-old woman with limited cutaneous systemic sclerosis and dementia presented with a 1-week history of nonproductive cough and fever.

**Diagnoses:**

Chest computed tomography (CT) showed bilateral consolidations, bronchial wall thickening, and mucus plugs, suggesting airway infection. However, both respiratory symptoms and CT findings worsened, and sputum and blood cultures remained negative. To differentiate SSc-ILD from viral infection, multiplex PCR testing (FilmArray) of a pharyngeal swab was performed, detecting RSV and confirming RSV pneumonia.

**Interventions:**

The patient was initially treated with intravenous ceftriaxone, then given prednisolone at 0.6 mg/kg/d after RSV was diagnosed.

**Outcomes:**

Her respiratory symptoms and CT findings improved, and she was discharged on day 16 with tapering corticosteroids.

**Lessons:**

Multiplex PCR is useful for detecting RSV pneumonia in patients with autoimmune diseases. Clinicians should be aware that RSV infection can occur in these patients and should consider RSV pneumonia as an important differential diagnosis of SSc-ILD.

## 1. Introduction

Respiratory syncytial virus (RSV) is a major cause of severe lower respiratory tract infections in infants.^[1]^ Recently, RSV has increasingly been recognized as a cause of severe pneumonia in adult patients through multiplex PCR testing.^[2–4]^ However, due to the lack of specific antiviral drugs and low awareness of RSV infection in adults, RSV pneumonia is likely underestimated in clinical practice. Systemic sclerosis (SSc) is an autoimmune disease characterized by skin thickening and progressive fibrosis of multiple organs.^[5,6]^ Interstitial lung disease (ILD) is one of the most serious complications of SSc and a major prognostic factor.^[6–8]^ As interstitial lung disease associated with systemic sclerosis (SSc-ILD) often requires long-term immunosuppressive therapy, distinguishing it from acute infections is essential for proper treatment planning.

Herein, we report a case of RSV pneumonia in an elderly patient with SSc-ILD. Multiplex PCR proved useful in detecting RSV pneumonia in this context. RSV infection should be considered an important differential diagnosis of SSc-ILD to avoid the inappropriate use of immunosuppressive agents, which may be harmful in cases of viral pneumonia.

## 2. Case presentation

**Figure 1.**
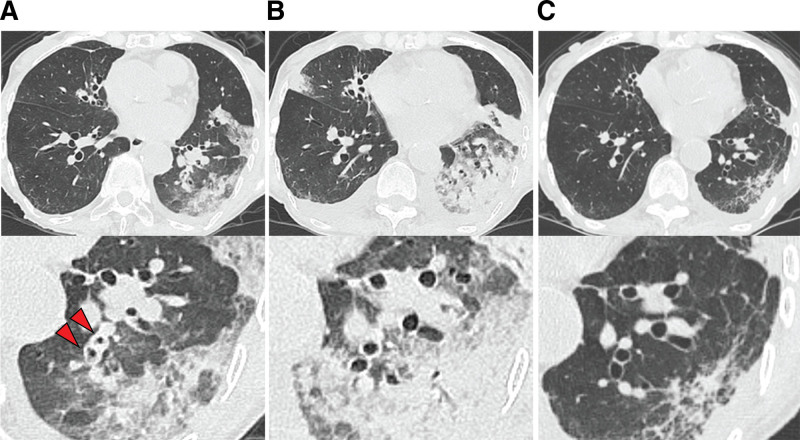
Chest CT on the day of admission (A) showed consolidations in the bilateral lower lobes and right middle lobe, along with bronchial wall thickening and mucus plugs (arrows indicate bronchial wall thickening). On hospital day 6 (B), consolidations in the bilateral lung fields had worsened. On hospital day 15 (C), after 9 days of treatment with prednisolone at 0.6 mg/kg/d, the consolidations had improved. CT = computed tomography.

**Figure 2.**
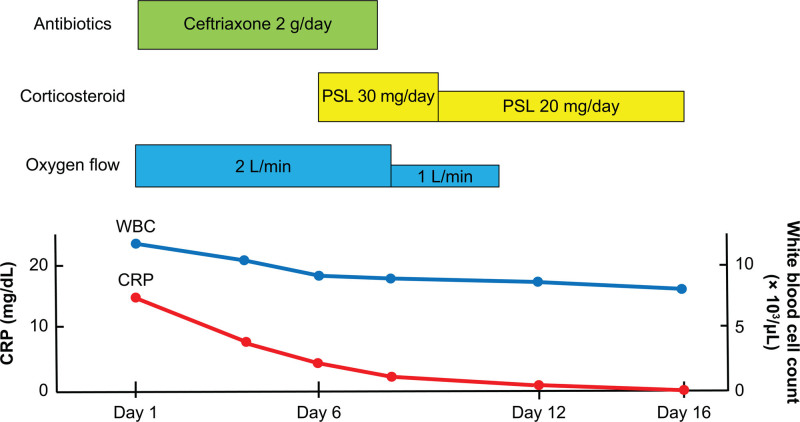
Clinical timeline following hospital admission. CRP = C-reactive protein; PSL = prednisolone.

An 85-year-old woman visited the outpatient clinic at our hospital with a chief complaint of nonproductive cough and fever for 1 week. She had a history of limited cutaneous systemic sclerosis without pulmonary involvement, such as ILD or pulmonary hypertension, and dementia. She had not been receiving corticosteroids or other immunosuppressive therapy. On physical examination, her body temperature was 37.8 °C, blood pressure 120/56 mm Hg, pulse rate 87 beats per minute, and oxygen saturation was 90% on room air. Lung sounds were clear to auscultation, and abdominal examination was unremarkable except for hepatosplenomegaly. Skin examination showed sclerodactyly consistent with systemic sclerosis, but no other cutaneous abnormalities. Laboratory tests revealed elevated serum C-reactive protein (18.34 mg/dL), white blood cell count (13,710/μL), and lactate dehydrogenase (236 U/L). PCR test for severe acute respiratory syndrome coronavirus 2, and rapid antigen tests for influenza virus and *Mycoplasma pneumoniae* from pharyngeal swabs, were negative. Chest computed tomography (CT) revealed bilateral consolidations, bronchial wall thickening, and mucus plugging (Fig. 1A), suggesting infection. She was hospitalized on the same day and treated with intravenous ceftriaxone. The clinical course after admission is shown in Figure 2. On day 6 of hospitalization, both respiratory failure and bilateral lung consolidation on chest CT had worsened (Fig. 1B). As sputum and blood cultures were negative, organizing pneumonia secondary to viral infection or SSc-ILD was suspected. Given that cultures remained negative and imaging findings were inconclusive, a multiplex PCR test (FilmArray; BioFire Diagnostics, Inc., a bioMérieux company, Salt Lake City) was performed on a pharyngeal swab, which subsequently detected RSV. She was diagnosed with RSV pneumonia, either alone or superimposed on SSc-ILD, and was started on prednisolone at 0.6 mg/kg/d. Her respiratory symptoms and CT findings subsequently improved (Fig. 1C), and she was discharged on day 16 with a reduced prednisolone dose of 0.3 mg/kg/d. Further tapering and discontinuation were planned in the outpatient clinic.

## 3. Discussion

RSV infection develops in 3% to 7% of adults over 65 years of age and is increasingly recognized as a significant cause of hospitalization and mortality in older adults.^[9]^ The mortality rate of RSV pneumonia in patients over 65 years has been reported to be as high as 50%.^[2]^ Risk factors for severe RSV infection include frailty, congestive heart failure, stroke, chronic kidney disease, chronic obstructive pulmonary disease, and immunosuppression.^[9]^ In our case, although the patient did not receive immunosuppressive agents or corticosteroids, older age and frailty due to SSc were risk factors for severe RSV infection, resulting in pneumonitis.

In this case, CT findings revealed not only consolidations suggestive of infection or organizing pneumonia, but also extensive bronchial wall thickening and mucus plugs, which pointed more toward an infectious etiology. A systematic review of CT findings in RSV pneumonia among adults aged 18 and older reported that nodules were the most frequent finding, followed by septal thickening and ground-glass opacities.^[10]^ In contrast, organizing pneumonia (27.6%) and bronchitis (26.5%), as seen in our case, were also reported, reflecting the diverse radiologic manifestations of RSV pneumonia. Although organizing pneumonia is a characteristic CT pattern in SSc-ILD, extensive bronchial wall thickening and mucus plugging are not typical.^[8]^ Thus, the CT findings in our case were more consistent with RSV pneumonia, either in isolation or superimposed on the organizing pneumonia pattern of SSc-ILD.

In addition, sputum and blood cultures were negative, there was no response to antibacterial agents, and RSV was detected via multiplex PCR, leading to a diagnosis of RSV pneumonia. RSV was identified using the multiplex PCR assay FilmArray, which detects 22 pathogens from nasopharyngeal swabs.^[4]^ These assays have high diagnostic performance, with sensitivities from 84% to 100% and specificities from 97.7% to 100%.^[3]^ They can simultaneously detect common respiratory pathogens, including *Mycoplasma pneumoniae*, influenza virus, and coronaviruses. In patients with autoimmune diseases, such as SSc, multiplex PCR is especially useful for distinguishing infection from autoimmune-associated ILD. In our case, treatment with corticosteroids alone was sufficient after identifying RSV. A previous study on hospitalized RSV pneumonia patients showed that short-term systemic corticosteroid use did not significantly affect viral load, shedding duration, or adverse effects.^[1]^ Currently, treatment options for RSV infection in adults remain limited and unstandardized. Although ribavirin is the only antiviral agent approved for RSV,^[3]^ it is not approved in Japan. In contrast, standard treatments for SSc-ILD include corticosteroids and immunosuppressive agents like cyclophosphamide and mycophenolate mofetil.^[5–7]^ Antifibrotic agents have also been used to slow lung function decline in SSc-ILD.^[11]^ Although our patient improved with corticosteroids alone, we cannot be certain whether RSV pneumonia alone or whether SSc-ILD also contributed to lung involvement based on the treatment response. However, given that initiating immunosuppressive therapy without excluding infection can worsen outcomes, confirming RSV through multiplex PCR was critical for guiding management.

Our patient responded well to corticosteroids alone once RSV pneumonia was diagnosed. However, no evidence is available to suggest that corticosteroids are effective against RSV itself, and the benefit may have reflected treatment of SSc-ILD in this case. Even so, RSV infection may have acted as a trigger for SSc-ILD, underscoring the importance of RSV prevention. An RSV vaccine has been approved for older adults and significantly reduces the risk of RSV-related lower respiratory tract disease.^[9]^ As differentiating RSV pneumonia from ILD associated with autoimmune diseases like SSc can be challenging, vaccination may serve as a key preventive strategy. We experienced a case of RSV pneumonia in a patient with systemic sclerosis. Clinicians should be aware that RSV infection can occur in patients with autoimmune diseases, and that multiplex PCR testing is a useful diagnostic tool. RSV pneumonia should be considered a key differential diagnosis of SSc-ILD.

## Author contributions

**Data curation:** Hiroki Wakabayashi, Eisuke Tanaka, Ryogo Ohashi, Kensuke Namba, Misa Iwayanagi, Hiromasa Sakurai, Yusuke Irie, Daiki Sakai, Kenta Takashima, Yu Murakami, Ai Nishida.

**Visualization:** Hiroki Wakabayashi.

**Writing – original draft:** Hiroki Wakabayashi.

**Writing – review & editing:** Kaichi Kaneko, Kazutoshi Isobe, Yasuo Matsuzawa.
